# The Potential Mediating Role of HDL-Cholesterol Concentrations in the Association between Physical Activity and Depression: A Nationwide Population-Based Study

**DOI:** 10.1016/j.cdnut.2026.107691

**Published:** 2026-04-11

**Authors:** Junyu Zhou, Bingqing Guan

**Affiliations:** Key Laboratory of Reproductive Health Diseases Research and Translation of Ministry of Education, Department of Medical Psychology, The First Affiliated Hospital, Hainan Medical University, , Haikou, Hainan Province, China

**Keywords:** physical activity, HDL-C, depression, mediation analysis, NHANES

## Abstract

**Background:**

Depression is a leading cause of global disability. Physical activity (PA) has been found to be associated with lower risk of depression, but the underlying biological mediating factors remain understudied. HDL cholesterol (HDL-C) is an important biomarker with neuroprotective properties, however, its potential mediating role in the relationship between PA and depression has not been explored.

**Objectives:**

Our study aimed to investigate the role of HDL cholesterol in the association between PA and depressive symptoms.

**Methods:**

A total of 16,535 adults from the NHANES (2009–2016) were analyzed. Depression was assessed with the Patient Health Questionnaire-9, and data on PA and HDL-C were collected for each participant. Multivariate logistic regressions, subgroup analysis, mediation analysis, and sensitivity analysis were performed.

**Results:**

After adjusting for covariates, individuals with sufficient PA had 57% lower prevalence of depression [odds ratio (OR): 0.43; 95% confidence interval (CI): 0.35, 0.53] compared with those reporting insufficient PA. Of note, each unit increase in HDL-C associated with 23% lower odds of depression (OR: 0.77; 95% CI: 0.65, 0.90). Mediation analysis suggested a small but statistically significant indirect effect of HDL-C in the association between PA and depressive symptoms (indirect effect: −0.03; 95% CI: −0.04, −0.01), accounting for 3.3% of the total effect.

**Conclusions:**

PA showed a protective association with depression through mediation of HDL-C, highlighting a metabolic pathway in the association between PA and mental health. Promoting PA and optimizing HDL-C concentration may help prevent depression at population levels.

## Introduction

Depression is a multifactorial mental disorder and ranks among the leading causes of disability worldwide [1]. According to the WHO, >280 million people experience depression each year [2]. Depression significantly reduces patients’ quality of life and imposes a huge socioeconomic burden [3,4]. The etiology of depression is related to multiple factors such as genetics, environment, and lifestyle [5]. Physical activity (PA), as a modifiable behavioral factor, has attracted much attention for its role in alleviating depressive symptoms and improving mental health [6]. A large body of studies have shown that PA can significantly reduce depressive symptoms, improve mood, and reduce the risk of relapse [7,8]. Many biomarkers have been shown to mediate the association between PA and depression. PA may alleviate depressive symptoms by reducing the inflammatory response [9]. PA could also reduce depression by enhancing neuroplasticity [10]. Brain-derived neurotrophic factor (BDNF), a key factor in the promotion of neuroplasticity, has also been suggested to potentially ameliorate depressive symptoms through regular PA [11].

HDL cholesterol (HDL-C) is an important biomarker in lipid metabolism, with both cardiovascular and neural protective effects [12]. Both cohort and cross-sectional studies have indicated that low HDL-C concentrations could elevate the risk of depression, anxiety, and stress-related disorders [13,14]. HDL-C may have a positive effect on brain health by lowering systemic inflammation and regulating oxidative stress [15]. Despite the progress in the abovementioned areas, no research has explicitly investigated the mediating effect of HDL-C in the association between PA and depression. Although HDL-C has attracted attention for its cardiovascular metabolic benefits and possible effects on brain health, its specific contribution to the protective effect of PA on mental health has not been fully verified. This problem is particularly prominent in large-scale nationwide population studies, which provide systematic and representative evidence.

Our study aims to fill this gap by exploring whether HDL-C plays a mediating role between PA and depressive symptoms in adults using a nationally representative sample. Findings of our work may help to enhance the comprehension of the mechanisms by which PA benefits mental health and provide novel insights for developing targeted strategies to promote both physical and psychological well-being.

## Methods

### Data source and participant selection

This research utilized data from the NHANES, an ongoing national program led by the National Center for Health Statistics (NCHS) to evaluate the health and nutritional status of the US population. NHANES implements an intricate, stratified, multiphase sampling strategy to guarantee data representativeness and uphold high standards of quality. Trained professionals carried out the survey and gathered comprehensive information, including demographics, socioeconomic status, dietary habits, and health-related factors. All participants gave informed consent before participation. The NCHS Research Ethics Committee evaluated and authorized the study design [16].

**FIGURE 1:**
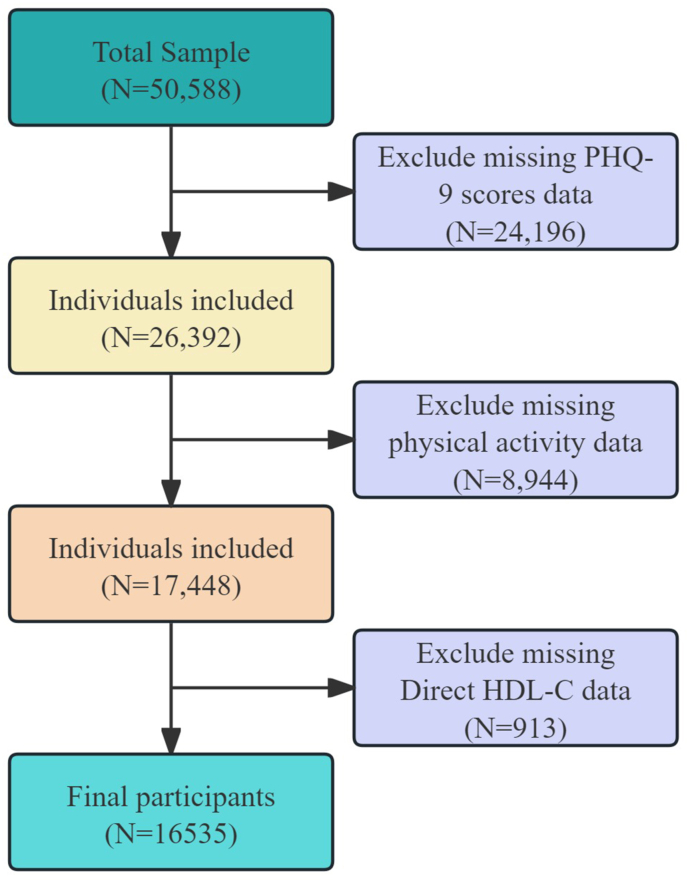
Flowchart of the study design and participants selection. HDL-C, HDL cholesterol; PHQ-9, Patient Health Questionnaire-9.

Our study used NHANES data from 2009 to 2016 and restricted the analytic sample to participants aged ≥20 y. We chose this age threshold to align with NHANES/Centers for Disease Control and Prevention (CDC) adult lipid reporting conventions and our prespecified age strata (20–39, 40–59, and ≥60 y) [[17], [18], [19]], rather than to redefine adulthood. The initial sample included 50,588 participants. Our study excluded several groups: participants without Patient Health Questionnaire-9 (PHQ-9) data (*n* = 24,196), participants missing PA data (*n* = 8944), and individuals lacking HDL-C test (*n* = 913). After applying these criteria, the final sample included 16,535 adults (Figure 1).

### Measurements

#### Depression

The PHQ-9 was used to assess depressive symptoms, which is a reliable tool for detecting and assessing the severity of depressive symptoms within the past 2 wk [20]. It is widely used due to its excellent validity, reliability, free access, and simplicity [21]. The PHQ-9 contains 9 items, and the symptoms of each item cover the criteria for diagnosing depression as defined in the fifth edition of the *Diagnostic and Statistical Manual of Mental Disorders* [22,23]. Each item adopts a 4-point score between 0 (not at all) and 3 (almost every day) according to the frequency of symptoms, and the overall score spans from 0 to 27 [24]. In our study, participants with a total PHQ-9 score of ≥10 were classified as having depressive symptoms [25], and the Cronbach’s α was 0.85, indicating good internal consistency [26].

#### HDL-C

Participants fasted for ≥8.5 h before blood sample collection. After qualified samples were selected, they were first subjected to a magnesium sulfate/dextran solution to create a water-soluble complex with non–HDL-C part to prevent it from participating in subsequent reactions. After adding relevant reagents, polyethylene glycol (PEG) cholesterol esterase converted HDL-C esters into HDL-C. PEG cholesterol oxidase then catalyzed the production of hydrogen peroxide, which reacted with 4-aminoantipyrine and HSDA to generate purple or blue pigments. These pigments were measured at 600 nm (secondary wavelength 700 nm), and the results were collected and recorded as millimoles per liter. Finally, the participants’ serum HDL-C values were obtained. More specific laboratory test information is available on the CDC website [27,28].

#### PA

PA was assessed with Global Physical Activity Questionnaire [27,29], which collected information on vigorous or moderate-intensity exercise, fitness routines, and recreational activities during a typical week. All of the above activities must last ≥10 min. The formula used to calculate total PA time per week is: Total PA time (minutes per week) = (2 × days of vigorous PA × average daily minutes of vigorous PA) + days of moderate PA × average daily minutes of moderate PA [30]. In our study, participants’ PA patterns were divided into insufficient (0 ≤ total PA time < 150 min) and sufficient (total PA time ≥150 min) based on United States PA recommendation guidelines [30].

### Covariates

Based on previous studies [31,32], the covariates in our study included: sex (male, female), age (20–39, 40–59, ≥60 y), educational level (less than high school graduate, high school graduate, some college or associate degree, college graduate or higher), race (Mexican American, other Hispanic, non-Hispanic White, non-Hispanic Black, other races), income level [measured by the family poverty income ratio (PIR): low income, PIR ≤1.3; middle income, 1.3 < PIR < 3.5; high income, PIR ≥3.5] [33], marital status (married, widowed, divorced, separated, never married, living with a partner), and BMI, which was grouped into normal and low weight (BMI <25.0 kg/m^2^), overweight (25.0 kg/m^2^ ≤ BMI <30.0 kg/m^2^), obesity class I (30.0 kg/m^2^ ≤ BMI <35.0 kg/m^2^), obesity class II and higher (BMI ≥35.0 kg/m^2^) [34]. Individuals who consumed a minimum of 12 glasses of any type of alcoholic beverage in any given year were considered drinkers. Smokers were included in the study if they had smoked ≥100 cigarettes over the course of their lifetime. Data on coronary artery disease, diabetes, and hypertension were obtained from self-reported medical histories of patients diagnosed by professional physicians. In addition, our study also included total cholesterol (TC, millimoles per liter) as a covariate because HDL-C is an important component of TC. Covariate selection was further informed by a literature-based directed acyclic graph (DAG), which was constructed to identify sufficient adjustment sets and avoid overadjustment by illustrating the hypothesized relationships among PA, HDL-C, depressive symptoms, and the selected covariates. The DAG and supporting references are provided in Supplemental Figure 1.

### Statistical analysis

To ensure the national representativeness, our study processed data using the weighting method officially recommended by NHANES. First, participants were divided into 2 groups based on their PHQ-9 score. The Mann-Whitney *U*-test was used to compare continuous variables with skewed distribution, and the chi-square test was used to compare differences in categorical variables between the 2 groups. To explore the association between PA and HDL-C and depression, we applied 3 multivariate logistic regression models to estimate the adjusted odds ratio (OR) and its 95% confidence interval (CI). Model 1 was a crude model; model 2 was adjusted for age, sex, race, education level, marital status, and family PIR; and model 3 was further adjusted for smoking, drinking, hypertension, BMI, diabetes, coronary artery disease, and TC based on model 2. In these models, PA was considered as a binary variable (sufficient or insufficient), whereas HDL-C was modeled primarily as a continuous variable to preserve information and maintain statistical efficiency. Quartile-based analyses (Q1, Q2, Q3, and Q4) were performed as secondary analyses to examine the robustness and potential dose–response pattern of the association across increasing HDL-C concentrations; both approaches are reported to allow comparison and enhance interpretability [35]. To evaluate the independent effects of PA and HDL-C on depression in different subgroups, our study also conducted subgroup analyses, including age group (20–39, 40–59, ≥60 y), sex (male, female), education level (less than high school graduate, high school graduate, some college or associate degree, college graduate or higher), race (Mexican American, other Hispanic, non-Hispanic White, non-Hispanic Black), family PIR (PIR <1.3, 1.3 < PIR <3.5, PIR ≥3.5), marital status (married/living with partner, widowed/divorced/separated/never married), BMI (BMI <25.0 kg/m^2^, 25.0 kg/m^2^ ≤ BMI <30.0 kg/m^2^, 30.0 kg/m^2^ ≤ BMI<35.0 kg/m^2^, BMI ≥35.0 kg/m^2^), drinking, smoking, and disease conditions (hypertension, diabetes, and coronary artery disease). Subsequently, the R “mediation” package was applied to analyze the mediation effects [36], estimating the indirect, direct, and total effects of PA on depression in both the crude model and the fully adjusted model. The mediation effect ratio of HDL-C was calculated by dividing the indirect effect by the total effect, and the 95% CI of the mediation ratio was estimated. Furthermore, we conducted sensitivity analyses of multivariate logistic regression models and mediating effects after excluding participants with missing values in any covariates. R (version 4.4.0; R Core Team, R Foundation for Statistical Computing, Vienna, Austria) was used to perform complete statistical analyses. A statistically significant finding was indicated by a 2-sided *P* value < 0.05.

## Results

### Characteristics of study participants

A total of 16,535 participants were included in our study, representing 115.7 million noninstitutionalized American adults. The mean age (SD) of the participants was 48.61 ± 18.45 y, with 49.02% being male. Among the participants, 1755 (10.6%) showed symptoms of depression. Individuals with depression showed a significantly lower level of PA compared with those without depression (*P* < 0.001). The serum concentration of HDL-C in participants with depression was lower than that in nondepressed individuals (*P* < 0.001). There were also significant differences in age group, sex, education level, race, PIR, marital status, BMI status, smoking status, coronary artery disease, and diabetes between participants with depression and those without depression (*P* < 0.001) (Table 1).

**TABLE 1:** Weighted characteristics of the study participants. (*n* = 16,535)

Characteristic	Total participants	Depression	Nondepression	*P* value
	(*n* = 16,535)	(*n* = 1755)	(*n* = 14,780)	
Weighted sample size	115,680,604	11,709,494	103,971,110	
Age (y), mean (SD)	48.61 (18.45)	49.04 (16.61)	48.56 (18.66)	0.209
Age group (y), *n* (%)				<0.001
20–40	5077 (32.2)	479 (28.2)	4598 (32.7)	
40–60	5296 (33.6)	695 (40.9)	4601 (32.7)	
≥60	5405 (34.3)	524 (30.9)	4881 (34.7)	
Sex, *n* (%)				<0.001
Male	8106 (49.0)	614 (35.0)	7492 (50.7)	
Female	8429 (51.0)	1141 (65.0)	7288 (49.3)	
Educational level, *n* (%)				<0.001
Less than high school graduate	4483 (28.4)	670 (39.5)	3813 (27.1)	
High school graduate	3647 (23.1)	403 (23.7)	3244 (23.1)	
Some college or associate degree	4469 (28.3)	470 (27.7)	3999 (28.4)	
College graduate or higher	3167 (20.1)	155 (9.1)	3012 (21.4)	
Race/ethnicity, *n* (%)				<0.001
Mexican American	2812 (17.0)	293 (16.7)	2519 (17.0)	
Other Hispanic	1888 (11.4)	264 (15.0)	1624 (11.0)	
Non-Hispanic White	6829 (41.3)	731 (41.7)	6098 (41.3)	
Non-Hispanic Black	3428 (20.7)	359 (20.5)	3069 (20.8)	
Other races	1578 (9.5)	108 (6.2)	1470 (9.9)	
Family poverty income ratio, *n* (%)				<0.001
<1.3	5413 (35.9)	891 (56.2)	4522 (33.5)	
1.3–3.5	5606 (37.1)	512 (32.3)	5094 (37.7)	
≥3.5	4080 (27.0)	183 (11.5)	3897 (28.8)	
Marital status, *n* (%)				<0.001
Married	8032 (50.9)	609 (35.9)	7423 (52.7)	
Widowed	1313 (8.3)	180 (10.6)	1133 (8.0)	
Divorced	1751 (11.1)	292 (17.2)	1459 (10.4)	
Separated	563 (3.6)	120 (7.1)	443 (3.1)	
Never married	2817 (17.9)	331 (19.5)	2486 (17.7)	
Living with partner	1296 (8.2)	164 (9.7)	1132 (8.0)	
BMI (kg/m^2^), mean (SD)	29.32 (7.11)	31.08 (8.54)	29.11 (6.89)	<0.001
BMI group (kg/m^2^), *n* (%)				<0.001
<25	4686 (28.7)	411 (23.8)	4275 (29.3)	
25–30	5294 (32.4)	462 (26.7)	4832 (33.1)	
30–35	3468 (21.2)	425 (24.6)	3043 (20.8)	
≥35	2896 (17.7)	432 (25.0)	2464 (16.9)	
Alcohol user, *n* (%)	11,352 (70.0)	1212 (70.2)	10,140 (70.0)	0.864
Smoker, *n* (%)	7278 (45.3)	1034 (60.1)	6244 (43.5)	<0.001
Hypertension, *n* (%)	6009 (36.4)	859 (49.1)	5150 (34.9)	<0.001
Diabetes mellitus, *n* (%)	2202 (13.3)	365 (20.8)	1837 (12.4)	<0.001
Coronary artery disease, *n* (%)	668 (4.3)	119 (7.1)	549 (3.9)	<0.001
TC (mmol/L), mean (SD)	4.96 (1.09)	5.07 (1.17)	4.95 (1.08)	<0.001
PHQ-9 score, mean (SD)	3.56 (4.58)	14.30 (4.01)	2.29 (2.50)	<0.001
Physical activity, *n* (%)				<0.001
Insufficient (<150 min/wk)	13,002 (78.6)	1617 (92.1)	11,385 (77.0)	
Sufficient (≥150 min/wk)	3533 (21.4)	138 (7.9)	3395 (23.0)	
HDL cholesterol (mmol/L), mean (SD)	1.35 (0.42)	1.31 (0.42)	1.36 (0.42)	<0.001

Abbreviations: PHQ-9, Patient Health Questionnaire-9; TC, total cholesterol.

### Associations of PA and HDL-C with depression

After adjusting for all covariates (model 3), individuals with sufficient PA had 57% lower odds of depression (OR: 0.43; 95% CI: 0.35, 0.53; *P* < 0.001) (Table 2). As a continuous variable (model 3), the odds of depression decreased by 23.0% for each unit increase in HDL-C (OR: 0.77; 95% CI: 0.65, 0.90; *P* = 0.001). In addition, compared with the lowest HDL-C serum concentration (Q1), participants with the highest HDL-C serum concentration (Q4) had a 27% lower likelihood (OR: 0.73; 95% CI: 0.61, 0.87; *P* < 0.001) (Table 2).

**TABLE 2:** Associations of physical activity and HDL cholesterol with depression in adults^1^

	Model 1			Model 2			Model 3		
	OR	95% CI	*P* value	OR	95% CI	*P* value	OR	95% CI	*P* value
Physical activity									
Insufficient (<150 min/wk)	Ref.			Ref.			Ref.		
Sufficient (≥150 min/wk)	0.28	(0.23, 0.34)	<0.001	0.39	(0.31, 0.47)	<0.001	0.43	(0.35, 0.53)	<0.001
HDL cholesterol									
As continuous	0.73	(0.64, 0.83)	<0.001	0.65	(0.56, 0.76)	<0.001	0.77	(0.65, 0.90)	0.001
Q1 (<1.06 mmol/L)	Ref.			Ref.			Ref.		
Q2 (1.06–1.29 mmol/L)	0.80	(0.70, 0.92)	0.001	0.77	(0.66, 0.90)	<0.001	0.85	(0.73, 1.00)	0.056
Q3 (1.29–1.58 mmol/L)	0.75	(0.65, 0.86)	<0.001	0.69	(0.59, 0.81)	<0.001	0.81	(0.69, 0.96)	0.016
Q4 (>1.58 mmol/L)	0.69	(0.60, 0.79)	<0.001	0.60	(0.51, 0.70)	<0.001	0.73	(0.61, 0.87)	<0.001
*P*-trend			<0.001			<0.001			<0.001

Abbreviations: CI, confidence interval; OR, odds ratio; Q, quartile; Ref, reference.

1 The associations of physical activity and HDL cholesterol with depression are presented as OR (95% CI). Model 1 did not adjust for any covariates. Model 2 adjusted for age, sex, race, education level, marital status, and the family poverty income ratio. Model 3 further adjusted for total cholesterol, smoking cigarettes, alcohol use, hypertension, BMI, hypertension, diabetes, and coronary artery disease based on model 2.

The results of PA and HDL-C were verified in sensitivity analysis after excluding participants with any missing values (Supplemental Table 1).

### Subgroup analysis

**FIGURE 2:**
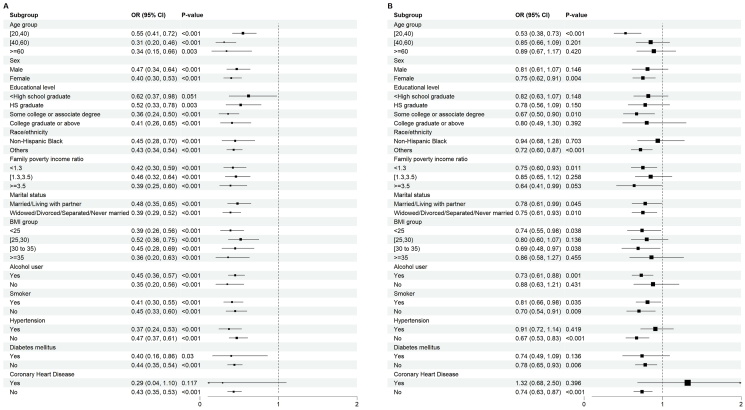
Subgroup analysis of the association between physical activity, HDL cholesterol, and depression. CI, confidence interval; OR, odds ratio.

The results showed that PA was associated with a lower risk of depression in all subgroups except those without coronary artery disease and less than high school education (Figure 2). Of note, the protective effect was significant for the age group 20 to 39 y, females, races other than non-Hispanic Black, low (PIR <1.3) or high family income (PIR >3.5), whether living with a partner or living alone, BMI <25 or 30 ≤ BMI <35 kg/m^2^, drinking, whether smoking or not, and people without hypertension, diabetes, or coronary artery disease (Figure 2). In addition, there was a significant interaction between age group and the relationship between PA and depression (*P*-interaction = 0.031).

### Mediation analysis

**FIGURE 3:**
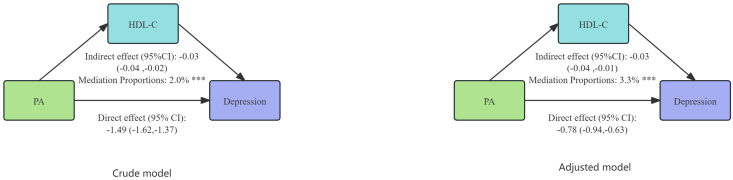
The mediation analysis of HDL-C in the association between PA and depression. ∗∗∗*P* < 0.001. CI, confidence interval; HDL-C, HDL cholesterol; PA, physical activity.

The analysis results showed that in the crude model, PA had a significant indirect effect on depression through HDL-C (indirect effect: −0.03; 95% CI: −0.04, −0.02), and the indirect effect accounted for 2.0% of the total effect (*P* < 0.001). After adjustment for all covariates, HDL-C accounted for a small proportion of the association between PA and depressive symptoms in the adjusted mediation model (indirect effect: −0.03; 95% CI: −0.04, −0.01), accounting for 3.3% of the total effect (Figure 3). After excluding missing values, the indirect effect was still significant (Supplemental Figure 2). In addition, the mediating effect of HDL-C was significant in people aged 20 to 59 y, females, races other than non-Hispanic Blacks, low (PIR <1.3) or high family income (PIR >3.5), individuals who were married or living with a partner, BMI <35 kg/m^2^, and people without hypertension, diabetes, or coronary artery disease (Supplemental Table 2).

## Discussion

Our study explored the mediating role of HDL-C in the relationship between PA and depressive symptoms using a nationwide sample of adults. We found that PA and HDL-C showed significant protective effects on depression, and HDL-C showed a modest indirect effect in the association between PA and depressive symptoms. Given the cross-sectional design, this finding should be interpreted cautiously and requires confirmation in longitudinal studies. In addition, subgroup analysis revealed differences in protective and mediating effects in specific populations (ages 20–59 y, females, those with BMI <35 kg/m^2^, and those without coronary artery disease, diabetes, and hypertension), providing a new perspective for understanding the complex interactions between PA, HDL-C, and depression.

Higher PA was associated with lower odds of depressive symptoms in our study, which is consistent with previous prospective evidence showing that physically active individuals are less likely to develop depression. A meta-analysis of prospective cohort studies by Schuch et al. [37] showed that higher levels of PA can reduce the risk of depression by 17%–41%. Previous prospective evidence suggests an inverse curvilinear dose–response association between PA and incident depression, with the greatest marginal benefits observed when moving from no activity to low-to-moderate activity levels [38]. A systematic review [39] found that adequate PA can reduce the risk of depression by 57% and further pointed out that PA may alleviate depressive symptoms through multiple mechanisms such as regulating neurotransmitters (such as serotonin and dopamine), enhancing the expression of neurotrophic factors (such as BDNF), and improving the function of the hypothalamus-pituitary-adrenal axis.

Moreover, higher concentrations of HDL-C were associated with a significantly lower risk of depression. The results of a cohort study [13] showed that for each unit increase in HDL-C, the risk of depression decreased by 12%, which was consistent with our study. The neuroprotective effect of HDL-C may be related to the following pathways: first, where HDL-C binds to paraoxonase-1 to clear oxidized low-density lipoprotein, thereby reducing oxidative damage to the central nervous system [40]. Additionally, HDL-C inhibits macrophage and microglial activation in hypercholesterolemia, lowering proinflammatory factor release and neuroinflammation [41].

Our study further suggested that HDL-C accounted for a small but statistically significant indirect effect in the association between PA and depressive symptoms, suggesting that PA may indirectly affect depressive symptoms by regulating lipid metabolism. Previous studies have demonstrated that HDL-C concentrations in healthy populations were significantly elevated after aerobic exercise [42,43]. The findings of this study extend to older demographics [44] and individuals afflicted with cardiovascular disease [45], where PA also led to a notable enhancement in functions of HDL-C. On the other hand, chronic inflammatory states have been shown to be closely related to the occurrence of depression [46]. PA may exert an antidepressant effect by dynamically regulating the inflammatory-immune balance. For example, regular exercise can reduce the concentrations of proinflammatory factors (such as IL-6 and TNF-α) and upregulate anti-inflammatory factors (such as IL-10) [47]. As mentioned above, HDL-C also reduces the risk of depression through an anti-inflammatory pathway. Given the cross-sectional design and the modest magnitude of the indirect effect, the mediating role of HDL-C should be interpreted with caution. Although the indirect effect is relatively small, recent methodological guidance also suggests that mediation results should not be evaluated solely by the proportion mediated, as this quantity may be unstable and should be interpreted alongside the size and potential clinical relevance of the indirect effect [48]. Thus, HDL-C may be regarded as one possible, but limited, pathway linking PA with depressive symptoms rather than a dominant mechanism.

Our findings also highlight the differential physiological and metabolic effects of HDL-C in different subpopulations. First, the mediating effect of HDL-C in the 20–59 y old population may be related to more active lipid metabolism and less age-related decline in HDL-C concentrations [49]. Young people generally have higher HDL-C concentrations and more efficient cholesterol transport, which may enhance the protective effect of HDL-C against depression. The significant mediation effect observed in females may be related to hormonal differences, especially the role of estrogen. Studies have shown that estrogen can positively regulate HDL-C concentrations, enhance its efflux and function, and enhance its anti-inflammatory and antioxidant functions [50], which may contribute to the stronger association of HDL-C with depression prevention in females. In addition, women generally have a higher incidence of depression [51], and estrogen can also promote the occurrence of depression [52], making the protective effect of HDL-C more significant in this subgroup.

Our study has several strengths, including a nationally representative sample; HDL-C is a laboratory indicator, and the data obtained are objective; multiple confounding factors such as age, sex, BMI, coronary artery disease, diabetes, and hypertension were fully controlled in the analysis, thereby enhancing the reliability of the conclusions. However, our study also has certain limitations. First, due to the cross-sectional data, it is difficult to determine the causal path relationship of HDL-C as a mediating variable, and the small indirect effect of HDL-C should therefore be interpreted cautiously. Second, PA and depression symptoms rely on self-reported data, which may be subject to memory bias or social desirability effects. Third, given the multifactorial nature of depressive symptoms, HDL-C is likely to represent only one of several possible pathways linking PA with depressive symptoms. Future longitudinal studies are needed to further clarify the temporal sequence and to verify the potential mediating role of HDL-C. Future studies should verify the mediating role of HDL-C through longitudinal design, and more objective assessments of depression, such as structured clinical interviews, are also warranted.

In conclusion, our study showed that HDL-C has a significant mediating role between PA and depressive symptoms. These findings provide a new perspective for exploring the metabolic mechanisms of depression and emphasize the importance of promoting PA and improving HDL-C concentrations in the prevention and treatment of depression.

## Author contributions

The authors’ responsibilities were as follows – JZ: wrote, reviewed, and edited the original draft, curated data, responsible for formal analysis, visualization, methodology, software, and conceptualization; BG: wrote, reviewed, and edited the manuscript, responsible for project administration, supervision, and conceptualization; and both authors read and approved the final manuscript.

## Data availability

Data in our study can be accessed directly from the NHANES website: https://wwwn.cdc.gov/nchs/nhanes/Default.aspx.

## Funding

The authors reported no funding received for this study.

## Conflict of interest

The authors report no conflicts of interest.
