# Supplementary material for: The Potential Mediating Role of HDL-Cholesterol Concentrations in the Association between Physical Activity and Depression: A Nationwide Population-Based Study
Source: Curr Dev Nutr. 2026 Apr 11;10(5):107691. doi: 10.1016/j.cdnut.2026.107691 (PMC13157156; doi:10.1016/j.cdnut.2026.107691)
Supplement: Multimedia component 1 [file mmc1.docx]

The potential mediating role of high-density lipoprotein cholesterol levels in the association between physical activity and depression: a nationwide population-based study

Junyu Zhou et al

| Table S1. Associations of physical activity and HDL with depression in adults without missing values ^a^. | | | | | | | | | |
| --- | --- | --- | --- | --- | --- | --- | --- | --- | --- |
|  | Model 1 | | | Model 2 | | | Model 3 | | |
|  | OR | 95% CI | P-value | OR | 95% CI | P-value | OR | 95% CI | P-value |
| PA |  |  |  |  |  |  |  |  |  |
| Insufficient (<150 min/week) | Ref. |  |  | Ref. |  |  | Ref. |  |  |
| Sufficient (≥150 min/week) | 0.28 | 0.23-0.34 | <0.001 | 0.39 | 0.31-0.47 | <0.001 | 0.43 | 0.35-0.53 | <0.001 |
| HDL-C |  |  |  |  |  |  |  |  |  |
| As continuous | 0.73 | 0.63-0.83 | <0.001 | 0.65 | 0.56-0.76 | <0.001 | 0.77 | 0.65-0.90 | 0.001 |
| Q1 (<1.06 mmol/L) | Ref. |  |  | Ref. |  |  | Ref. |  |  |
| Q2 (1.06-1.29 mmol/L) | 0.82 | 0.71-0.95 | 0.009 | 0.78 | 0.67-0.91 | 0.001 | 0.85 | 0.73-1.00 | 0.056 |
| Q3 (1.29-1.58 mmol/L) | 0.78 | 0.67-0.91 | 0.001 | 0.70 | 0.60-0.82 | <0.001 | 0.81 | 0.69-0.96 | 0.016 |
| Q4 (>1.58 mmol/L) | 0.68 | 0.59-0.80 | <0.001 | 0.60 | 0.51-0.71 | <0.001 | 0.73 | 0.61-0.87 | <0.001 |
| P for trend |  |  | <0.001 |  |  | <0.001 |  |  | <0.001 |
| Note: CI, confidence interval; OR, odds ratio; PA, physical activity; HDL-C, high-density lipoprotein cholesterol. ^a^ The associations of physical activity and HDL-C with depression are presented as ORs (95% CI). Model 1 did not adjust for any covariates. Model 2 adjusted for age, sex, race, education level, marital status, the ratio of family income to poverty. Model 3 further adjusted for total cholesterol, smoking cigarettes, alcohol use, hypertension, BMI, hypertension, diabetes, coronary heart disease based on Model 2. | | | | | | | | | |

| Table S2. Subgroup mediation analysis of HDL in the association between PA and depression in adults. | | | |
| --- | --- | --- | --- |
|  | Indirect effect (95% CI) | Mediation proportions | P-value |
| Age group |  |  |  |
| [20,40) | -0.03 (-0.06,-0.01) | 5.74% | 0.012 |
| [40,60) | -0.03 (-0.07,-0.01) | 3.37% | 0.024 |
| ≥60 | -0.01 (-0.03,0.02) | 1.47% | 0.430 |
| Sex |  |  |  |
| Male | -0.00 (-0.02,0.01) | 0.79% | 0.560 |
| Female | -0.04 (-0.07,-0.02) | 3.80% | <0.001 |
| Educational level |  |  |  |
| <High school graduate | -0.00 (-0.01,0.01) | 0.17% | 0.744 |
| HS graduate | -0.01 (-0.05,0.00) | 2.42% | 0.120 |
| Some college or associate degree | -0.05 (-0.08,-0.02) | 4.84% | <0.001 |
| College graduate or above | -0.02 (-0.06,0.01) | 4.51% | 0.120 |
| Race/ethnicity |  |  |  |
| Non-Hispanic Black | -0.00 (-0.02,0.02) | 0.81% | 0.652 |
| Others | -0.03 (-0.05,-0.01) | 3.99% | <0.001 |
| Family poverty income ratio |  |  |  |
| <1.3 | -0.03 (-0.07,0.00) | 2.68% | 0.036 |
| [1.3,3.5) | -0.00 (-0.03,0.01) | 1.12% | 0.350 |
| ≥3.5 | -0.03 (-0.06,-0.01) | 6.11% | 0.004 |
| Marital status |  |  |  |
| Married/Living with partner | -0.03 (-0.05,-0.01) | 5.82% | 0.002 |
| Widowed/Divorced/Separated/Never married | -0.01 (-0.04,0.00) | 1.69% | 0.084 |
| BMI group |  |  |  |
| <25 | -0.02 (-0.06,0.00) | 3.59% | 0.038 |
| [25,30) | -0.02 (-0.06,0.00) | 4.44% | 0.040 |
| [30 to 35) | -0.02 (-0.06,0.00) | 3.43% | 0.032 |
| ≥35 | -0.00 (-0.03,0.02) | 0.35% | 0.760 |
| Alcohol user |  |  |  |
| Yes | -0.03 (-0.05,-0.01) | 4.11% | <0.001 |
| No | -0.00 (-0.02,0.01) | 0.93% | 0.320 |
| Smoker |  |  |  |
| Yes | -0.03 (-0.05,-0.01) | 2.75% | 0.010 |
| No | -0.02 (-0.04,0.00) | 3.27% | 0.034 |
| Hypertension |  |  |  |
| Yes | -0,02 (-0.04,0.01) | 1.70% | 0.140 |
| No | -0.02 (-0.04,-0.01) | 4.25% | 0.004 |
| Diabetes mellitus |  |  |  |
| Yes | -0.01 (-0.07,0.01) | 1.83% | 0.344 |
| No | -0.02 (-0.04,-0.01) | 3.25% | <0.001 |
| Coronary Heart Disease |  |  |  |
| Yes | 0.01 (-0.07,0.14) | -1.58% | 0.758 |
| No | -0.02 (-0.04,-0.01) | 3.56% | <0.001 |
| Note: CI, confidence interval; OR, odds ratio; BMI, body mass index. Mediation model adjusted for age, sex, race, education level, marital status, the ratio of family income to poverty, total cholesterol, smoking cigarettes, alcohol use, hypertension, BMI, hypertension, diabetes, coronary heart disease. | | | |


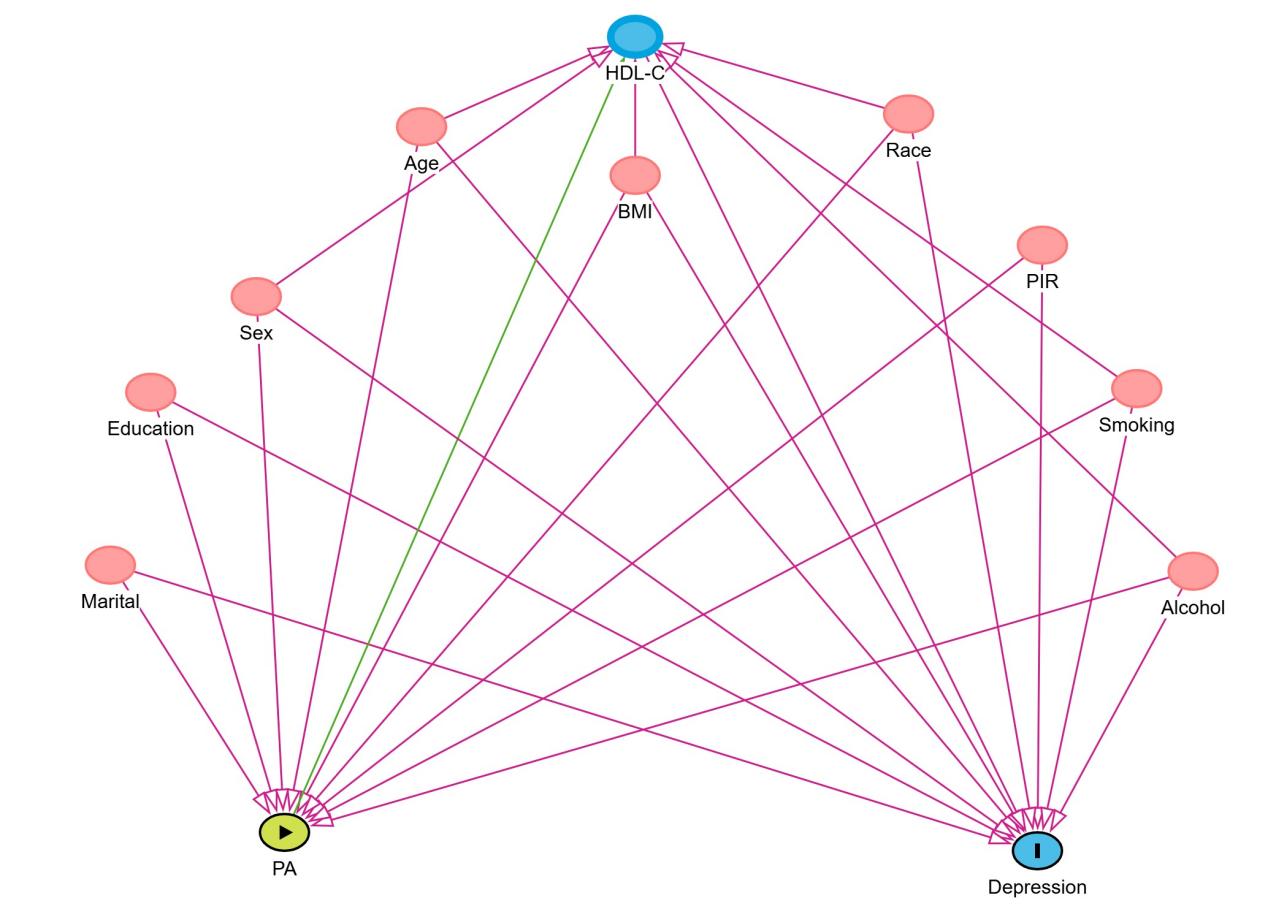


**Figure S1:** The DAG (Directed acyclic graph) depicts the hypothesized relationships among physical activity, HDL-C, depressive symptoms, and the included covariates. It was constructed based on prior literature to support the rationale for covariate selection. Representative references supporting the retained pathways are provided below [1-22].


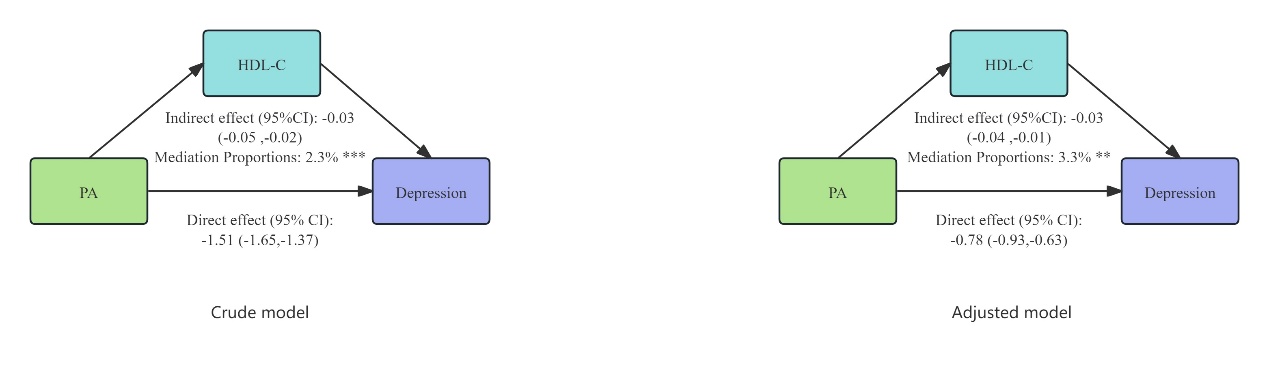


**Figure S2:** The mediation analysis of HDL-C in the association between PA and depression after excluding participants with missing values in covariates

Note: **<0.01; ***<0.001

1. Kelley, G.A. and K. Kelley, *Aerobic exercise and HDL2-C: a meta-analysis of randomized controlled trials.* Atherosclerosis, 2006. **184**(1): p. 207-215.

2. Pearce, M., et al., *Association between physical activity and risk of depression: a systematic review and meta-analysis.* JAMA psychiatry, 2022. **79**(6): p. 550-559.

3. Wei, Y.-G., et al., *Cholesterol and triglyceride levels in first-episode patients with major depressive disorder: A meta-analysis of case-control studies.* Journal of Affective Disorders, 2020. **266**: p. 465-472.

4. Caspersen, C.J., M.A. Pereira, and K.M. Curran, *Changes in physical activity patterns in the United States, by sex and cross-sectional age.* Medicine & science in sports & exercise, 2000. **32**(9): p. 1601-1609.

5. Wilson, P.W., et al., *Determinants of change in total cholesterol and HDL-C with age: the Framingham Study.* Journal of gerontology, 1994. **49**(6): p. M252-M257.

6. Kessler, R.C., et al., *Age differences in major depression: results from the National Comorbidity Survey Replication (NCS-R).* Psychological medicine, 2010. **40**(2): p. 225-237.

7. Davis, C., et al., *Sex difference in high density lipoprotein cholesterol in six countries.* American journal of epidemiology, 1996. **143**(11): p. 1100-1106.

8. Labaka, A., et al., *Biological sex differences in depression: a systematic review.* Biological research for nursing, 2018. **20**(4): p. 383-392.

9. Patel, N.A., et al., *Racial/ethnic disparities and determinants of sufficient physical activity levels.* Kansas journal of medicine, 2022. **15**(2): p. 267.

10. Gama, R., A. Elfatih, and N. Anderson, *Ethnic differences in total and HDL cholesterol concentrations: Caucasians compared with predominantly Punjabi Sikh Indo-Asians.* Annals of clinical biochemistry, 2002. **39**(6): p. 609-611.

11. Riolo, S.A., et al., *Prevalence of depression by race/ethnicity: findings from the National Health and Nutrition Examination Survey III.* American journal of public health, 2005. **95**(6): p. 998-1000.

12. Stalsberg, R. and A.V. Pedersen, *Are differences in physical activity across socioeconomic groups associated with choice of physical activity variables to report?* International journal of environmental research and public health, 2018. **15**(5): p. 922.

13. Lorant, V., et al., *Socioeconomic inequalities in depression: a meta-analysis.* American journal of epidemiology, 2003. **157**(2): p. 98-112.

14. Trost, S.G., et al., *Correlates of adults’ participation in physical activity: review and update.* Medicine & science in sports & exercise, 2002. **34**(12): p. 1996-2001.

15. Yan, X.Y., et al., *Marital status and risk for late life depression: a meta-analysis of the published literature.* Journal of International Medical Research, 2011. **39**(4): p. 1142-1154.

16. Rashid, S. and J. Genest, *Effect of obesity on high‐density lipoprotein metabolism.* Obesity, 2007. **15**(12): p. 2875-2888.

17. Luppino, F.S., et al., *Overweight, obesity, and depression: a systematic review and meta-analysis of longitudinal studies.* Archives of general psychiatry, 2010. **67**(3): p. 220-229.

18. Kaczynski, A.T., et al., *Smoking and physical activity: a systematic review.* American journal of health behavior, 2008. **32**(1): p. 93-110.

19. Luger, T.M., J. Suls, and M.W. Vander Weg, *How robust is the association between smoking and depression in adults? A meta-analysis using linear mixed-effects models.* Addictive behaviors, 2014. **39**(10): p. 1418-1429.

20. Dodge, T., P. Clarke, and R. Dwan, *The relationship between physical activity and alcohol use among adults in the United States: a systematic review of the literature.* American Journal of Health Promotion, 2017. **31**(2): p. 97-108.

21. Brien, S.E., et al., *Effect of alcohol consumption on biological markers associated with risk of coronary heart disease: systematic review and meta-analysis of interventional studies.* Bmj, 2011. **342**.

22. Li, J., et al., *Effect of alcohol use disorders and alcohol intake on the risk of subsequent depressive symptoms: a systematic review and meta‐analysis of cohort studies.* Addiction, 2020. **115**(7): p. 1224-1243.
